# Prognostic implications of dual tracer PET/CT: PSMA ligand and [^18^F]FDG PET/CT in patients undergoing [^177^Lu]PSMA radioligand therapy

**DOI:** 10.1007/s00259-020-05160-8

**Published:** 2020-12-18

**Authors:** Kerstin Michalski, Juri Ruf, Christian Goetz, Anna Katharina Seitz, Andreas K. Buck, Constantin Lapa, Philipp E. Hartrampf

**Affiliations:** 1 Department of Nuclear Medicine, Faculty of Medicine, Medical Center-University of Freiburg, Freiburg, Germany; 2 Department of Urology and Paediatric Urology, University Hospital Würzburg, Würzburg, Germany; 3 Department of Nuclear Medicine, University Hospital Würzburg, Würzburg, Germany; 4 Nuclear Medicine, Medical Faculty, University of Augsburg, Augsburg, Germany

**Keywords:** PSMA, FDG, PET/CT, Prostate cancer, Radioligand therapy

## Abstract

**Background:**

Prostate-specific membrane antigen (PSMA)-targeted radioligand therapy (RLT) with ^177^Lu-labeled PSMA ligands has achieved remarkable results in advanced disease stages of metastatic castration-resistant prostate cancer (mCRPC). However, not all patients benefit from this therapy. Different treatment responses could be explained by tumor heterogeneity triggered by progression and the number of prior treatments. PSMA-negative lesions can be missed on PSMA ligand PET/CT, which subsequently results in an underestimation of tumor burden. Conversely, high FDG uptake may also be an indicator of tumor aggressiveness and thus a poor prognostic marker for response to RLT and overall survival (OS). The aim of this analysis was to investigate the prognostic value of combined PSMA ligand PET/CT and [^18^F]fluorodeoxyglucose (FDG) PET/CT for outcome prediction in patients undergoing RLT.

**Materials and methods:**

This bicentric analysis included 54 patients with mCRPC who underwent both FDG and PSMA ligand PET/CT imaging before RLT. In all patients, the pattern of PSMA ligand and FDG uptake was visually assessed. Patients with at least one FDG-positive, but PSMA-negative (FDG+/PSMA−) lesions were compared to patients without any FDG+/PSMA− lesions. A log-rank analysis was used to assess the difference in OS between subgroups.

**Results:**

Median OS was 11 ± 1.8 months (95% CI 7.4–14.6). A significantly lower OS (*p* < 0.001) was found in patients with at least one FDG+/PSMA− lesion at baseline PET/CTs (*n* = 18) with a median OS of 6.0 ± 0.5 months (95% CI: 5.0–7.0 months). In comparison, patients without any FDG+/PSMA− lesions (*n* = 36) had a median OS of 16.0 ± 2.5 months (95% CI: 11.2–20.8 months).

**Conclusion:**

FDG+/PSMA− lesions are a negative predictor of overall survival in patients with mCRPC undergoing RLT. However, it remains to be determined if patients with FDG+/PSMA− lesions should be excluded from PSMA RLT.

**Supplementary Information:**

The online version contains supplementary material available at 10.1007/s00259-020-05160-8.

## Introduction

In advanced prostate cancer, tumor progression under androgen deprivation marks the transition from a hormone-sensitive to a castration-resistant stage of disease [1]. In metastatic castration-resistant prostate cancer (mCRPC), several classes of medical treatment have successfully prolonged survival, including next-generation androgen receptor signaling inhibitors such as abiraterone and enzalutamide [2, 3], or chemotherapy with docetaxel and cabazitaxel [4] [5] [6]. Additionally, prostate-specific membrane antigen- (PSMA) targeted radioligand therapy (RLT) with [^177^Lu]-labeled PSMA-ligands has achieved remarkable results in advanced disease stages [7]. The most important selection criterion for RLT is a high expression of PSMA of the tumor as assessed by adequate tracer uptake on PSMA ligand PET/CT [8]. Nevertheless, the patient group scheduled for PSMA-directed RLT is most likely heterogeneous both in terms of prior treatment as well as in terms of tumor biology. Parameters which may help in predicting and optimizing response rates to RLT are under investigation, such as additional assessment of tracer uptake on [^18^F]fluorodeoxyglucose (FDG) PET/CT that appears to be useful for the detection of more aggressive disease [9, 10]. In line with this, the semiquantitative measurement of FDG uptake at baseline FDG PET/CT appears to deliver independent prognostic information on overall survival (OS) in mCRPC [11]. In patients undergoing RLT with [^177^Lu]Lu-PSMA 617, a high baseline FDG uptake was associated with higher Gleason scores and poorer progression-free survival [12]. As tumor PSMA expression can decrease or get lost during several lines of treatment, additional FDG PET/CT scanning may play an important role in the detection of such lesions [13] [14]. Lesions showing increased FDG-uptake but no relevant uptake on PSMA ligand PET (FDG+/PSMA−) have been used as an exclusion criterion prior to PSMA RLT in a recent single-centre, single-arm phase 2 study with [^177^Lu]Lu-PSMA 617 therapy in patients with mCRPC (LuPSMA Trial) [14]. Whereas encouraging results could be shown for patients receiving RLT [14, 15], the subjects excluded from the trial due to FDG+/PSMA− lesions had a very poor OS under standard of care [16].

The aim of this bi-centric retrospective study was to evaluate the rate of FDG+/PSMA− lesions on PSMA ligand and FDG PET/CT in patients undergoing RLT and their prognostic implications. Therefore, dual FDG and PSMA ligand PET/CT scans before RLT were analyzed and the results correlated to OS.

## Patients and methods

### Patient cohort

Searching the databases of the University Hospital Würzburg for the period between December 2018 and February 2020 and that of the University Hospital Freiburg for the period between August 2018 and December 2019, all consecutive patients who underwent at least one cycle of RLT as systemic treatment for mCRPC were identified. PSMA ligand PET/CT was performed to assess eligibility for PSM-directed RLT. In case of adequate PSMA expression, the patients routinely underwent a subsequent FDG PET/CT scan to assess the presence of PSMA-negative metastases. In case of PSMA-negative metastases, PSMA RLT was only offered if the majority of metastases was PSMA-positive, and if PSMA RLT was the last therapeutic option.

Time intervals between PET/CT scans and first administration of RLT were as follows: PSMA ligand PET/CT and FDG PET/CT 24.9 ± 18.0 days (range: 1–68); PSMA ligand PET/CT and first cycle of RLT 35.6 ± 20.9 days (range: 2–85).

The study was approved by the local ethics committees (Freiburg: protocol no. 251/17; Würzburg: protocol no. 20190815 01).

### Imaging and treatment protocol

Whole-body PET scans were acquired using a PET/CT scanner with either full-dose contrast-enhanced diagnostic CT (PSMA ligand) or low-dose CT (FDG) for attenuation correction and anatomical co-registration. Both PET/CT studies were performed on two separate days. A detailed description of the imaging protocols can be found in the supplementary material. Standardized institutional protocols for RLT work-up were applied. In-house labeling was carried out for [^177^Lu]-labeled PSMA ligands ([^177^Lu]Lu-PSMA I&T (Würzburg), and [^177^Lu]Lu-PSMA 617 (Freiburg), which are considered comparable in their efficacy [8]. The standard PSMA RLT protocol consisted of infusion of 6.0 GBq of the radioligand every 6–8 weeks with up to 4 cycles depending on response to treatment.

### Image analysis

**Fig. 1:**
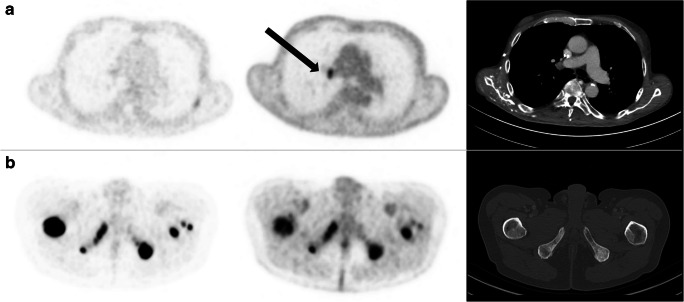
Corresponding axial slices of [^**18**^F]PSMA-1007 PET (first column), FDG PET (second column), and CT (third column). **a** 72-year-old patient with FDG+/PSMA- right hilar lymph node metastasis (black arrow; histologically proven metastasis from prostate cancer). **b** 65-year old patient with concordant FDG+/PSMA+ bone metastases. Fixed inverse gray-scale are displayed with SUV window setting from 0 to 10 ([^**18**^F]PSMA-1007 PET) and 0 to 5 (FDG PET), respectively

PET/CT images were retrospectively analyzed using commercial software packages (Würzburg: Syngo.via; VB30A, Siemens Healthcare, Erlangen, Germany; Freiburg: IMPAX EE; Agfa Health Care, Bonn, Germany). All lesions with non-physiological, higher uptake of the PSMA ligand or FDG than the physiological background were rated as PSMA− or FDG-positive, respectively. No size threshold was defined for positive lesions. Images were visually evaluated independently by two nuclear medicine specialists with at least 1-year experience in PET/CT reading using two categories: presence of one or more discordant lesions (FDG-positive but PSMA-negative) or absence of discordant FDG+/PSMA− lesions. These visual categories were established and validated in Freiburg by two readers (KM and CG) using the first 10 included patients. Interobserver agreement in the training phase was substantial, as indicated by Cohens *κ* = 0.78, based on Landis and Koch criteria [17]. Figure 1 gives examples of the categories. Furthermore, the number of discordant lesions was noted (1 lesion, ≤ 3 lesions, or ≥ 4 lesions). The extent of metastases on PSMA ligand PET/CT was classified following the PROMISE Classification [18] with modifications: low tumor burden (≤ 3 metastases), intermediate tumor burden (> 3 but < 10 metastases), high tumor burden (≥ 10 metastases), and diffuse bone marrow involvement.

### Statistical analysis

Statistical analyses were performed using SPSS software ver. 26.0 (IBM, Armonk, NY, USA). Descriptive data are presented as mean ± standard deviation and range. Survival data are analyzed by Kaplan-Meier curves and log-rank comparison. Univariate cox regression for continuous variables was applied. Multivariable analysis was undertaken for stratification of probable prognostic markers. OS was calculated starting with the first cycle of RLT and is presented as median ± standard error and 95% confidence interval [CI]. For statistical comparison between subgroups, an unpaired *t* test was performed. A *p* value less than 0.05 was considered statistically significant.

## Results

### Patients’ characteristics

Fifty-four patients (Würzburg: *n* = 26; Freiburg: *n* = 28) who underwent dual tracer PET/CT before at least one cycle of PSMA-targeted RLT were included in this analysis. Detailed characteristics of all included patients are given in Table 1. Mean patient age was 71.4 ± 8.5 years (range: 52–90). Mean time interval since initial diagnosis of prostate cancer and imaging was 7.7 ± 5.6 years (range: 1.7–26.3) and median initial Gleason score was 8 (range: 5–10, *n* = 48; unknown in six patients). Performance status according to Eastern Cooperative Oncology Group (ECOG) ranged from 0 to 3 (0: 52%; 1: 37%; 2: 9%; 3: 2%). Almost all patients (*n* = 52, 96%) presented with bone metastases. Other frequent sites of metastatic spread included lymph nodes (*n* = 33, 61%), the liver (*n* = 13, 24%), and the lungs (*n* = 8, 15%). At least one visceral metastasis (liver, lung, kidney, adrenal gland, testis, tumor in the (retro-) peritoneum, pleura, or leptomeningeal carcinomatosis) was found in 23 patients (53%). A low or intermediate tumor burden was found in two patients (4%) each. The majority (*n* = 41; 76%) of patients presented with a high tumor burden. Nine patients showed a diffuse bone marrow involvement (16%). More than two thirds of patients (69%) had been previously treated with at least one line of chemotherapy (docetaxel: *n* = 37; cabazitaxel: *n* = 12). Two patients had already undergone two cycles of RLT prior to the current staging more than 1 year before. The median number of systemic treatment lines before RLT was 3.5 (range: 2–6). Serum PSA level was 450 ± 916 ng/ml (range: 0.07–5000) at first cycle of RLT. In total, 139 cycles of RLT were administered with a mean activity of 5.9 ± 0.6 GBq (range: 1.9–6.4).

**Table 1:** Patient characteristics of all patients and depending on sub-groups

	All patients (*n* = 54)	FDG+/PSMA- (*n* = 18)	No FDG+/PSMA- (*n* = 36)
Age (years)	71.4 (52–90)	70.4 (58–82)	71.9 (52–90)
Time since diagnosis of prostate cancer (years)	7.7 (1.7–26.3)	6.3 (1.8–11.6)	8.4 (1.7–26.3)
Gleason score	8 (5–10)*	8 (7–10)~	8 (5–10)^#^
PSA (ng/ml)	450 (0.07–5000)	543 (0.07–5000)	404 (5–2650)
ECOG	0–3	0–3	0–2
Sites of disease:	*n* (patients)	*n* (patients)	*n* (patients)
Prostate/local	16	4	12
Lymph node	33	12	21
Bone	52	18	34
Liver	13	9	4
Lung	8	4	4
Other	9	5	4
Previous treatment:	*n* (patients)	*n* (patients)	*n* (patients)
Prostatectomy	28	9	19
Radiotherapy to prostate/ prostate bed	31	13	18
ADT	54	18	36
Abiraterone	44	15	29
Enzalutamide	29	9	20
Docetaxel	37	13	24
Cabazitaxel	12	3	9
[^223^Ra]Dichloride	8	3	5
Selective internal radiation therapy	2	0	2
PSMA RLT	2	1	1
Prostvac	1	1	0
Estramustin	1	0	1
Median lines of treatment before RLT	3.5 (2–6)	3.5 (2–6)	3.5 (2–6)
Number of RLT cycles	*n* (patients)	*n* (patients)	*n* (patients)
1 cycle	54	18	36
2 cycles	44	12	32
3 cycles	26	5	21
4 cycles	15	1	14

*PSA* prostate specific antigen, *ECOG* performance status according to Eastern Cooperative Oncology Group, *ADT* androgen deprivation therapy, *PSMA RLT* prostate-specific membrane antigen-targeted radioligand therapy, other sites of disease: kidney, adrenal gland, testis, tumor in the (retro-) peritoneum, pleura, leptomeningeal carcinomatosis; unknown in *n = 6, ~ *n* = 1, ^#^*n* = 5

### Disease group classification

Discordant lesions were found in 18 patients. FDG+/PSMA− lesions were seen mostly in the skeleton (*n* = 8) and the liver (*n* = 8), two patients showed discordant uptake in lymph nodes, one patient had a discordant prostate lesion, and a one patient had discordant peritoneal tumor in the pelvis. It is possible that patients had more than one “organ system” affected (e.g., bone and liver metastases in one patient as well as bone metastases, liver metastases, and a peritoneal tumor in the pelvis in another patient). In 7 patients, only one FDG+/PSMA− lesion was found, 8 patients had ≤ 3 discordant lesions, and in 3 patients, ≥ 4 FDG+/PSMA− lesions were detected. Patients with discordant lesions had a high tumor burden on PSMA ligand PET/CT (*n* = 15, 83%), and three patients even had a diffuse bone marrow involvement (17%).

Regarding the characteristics of the 18 subjects with one or more FDG+/PSMA− lesions (Table 1), these patients received significantly fewer cycles of RLT (2.0 ± 0.9, range: 1–4) than the other patients (2.9 ± 1.1, range: 1–4; *p* = 0.005). No difference was found for median Gleason score (8 for both subgroups). In addition, serum PSA levels (543 ± 1258, range: 0.07–5000 ng/ml vs. 404 ± 608, range: 5–2650 ng/ml; *p* = 0.608), time since diagnosis of prostate cancer (6.3 ± 3, range: 1.8–11.6 years vs. 8.4 ± 6.6, range 1.7–26.3 years, *p* = 0.209), pre-treatment with docetaxel (72.2% vs. 66.7%; *p* = 0.685), and ECOG performance status (range: 0–3 vs. 0–2; *p* = 0.2) did not differ significantly between subgroups.

### Prognostic factors

**Fig. 2:**
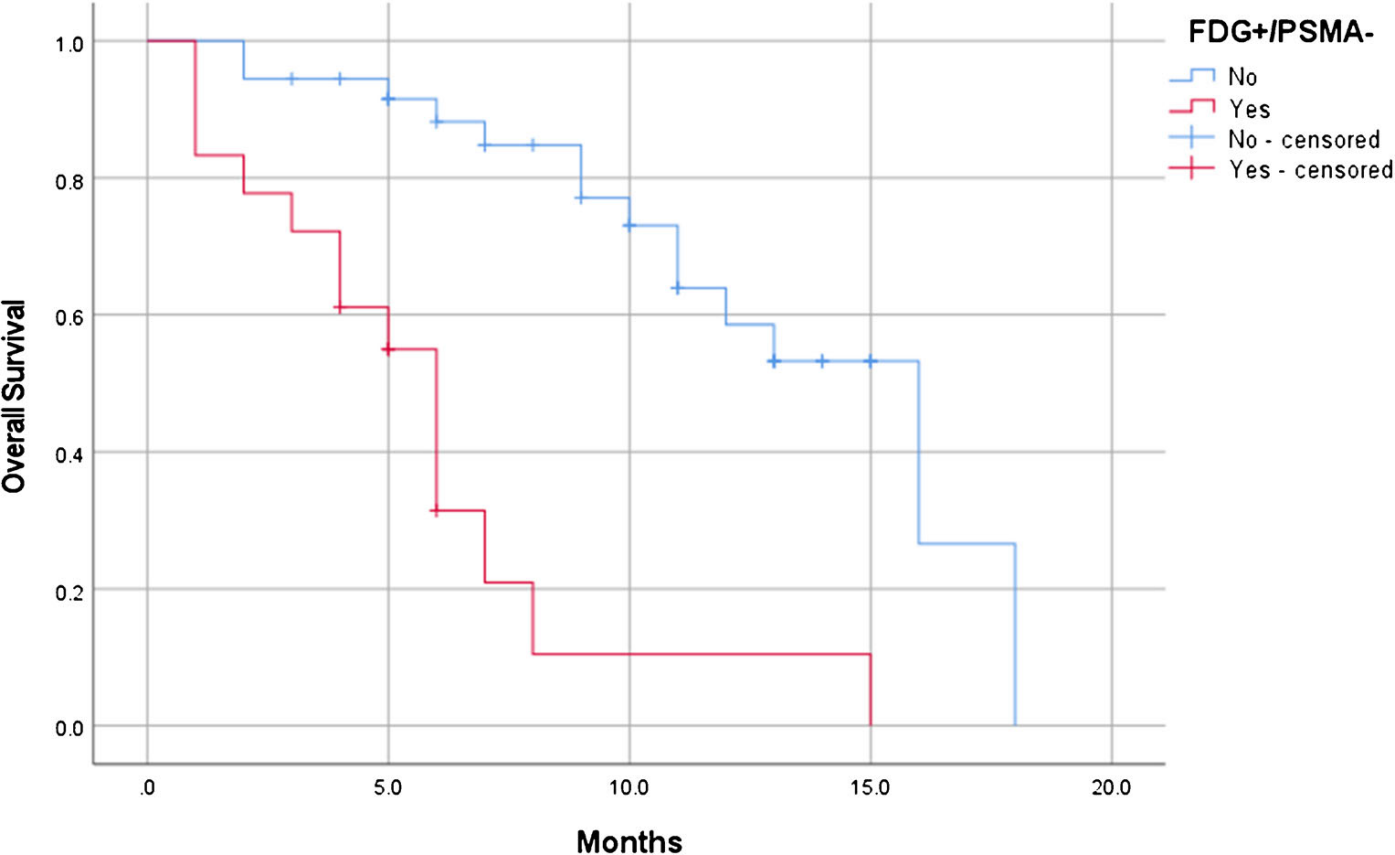
Kaplan-Meier curves of median overall survival (OS) of patients with FDG+/PSMA- lesions. These patients (*n* = 18, red line) had an OS of 6.0 ± 0.5 months (95% CI 5.0–7.0 months), whereas the other patients (*n* = 36, blue line) showed an OS of 16.0 ± 2.4 months (95% CI 11.2–20.8 months; *p* < 0.001)

Median OS of all patients (*n* = 54) was 11 ± 1.8 months (95% CI 7.4–14.6 months). Patients with one or more FDG+/PSMA− lesions (*n* = 18/54) had a significantly shorter OS (6.0 ± 0.5 months; 95% CI 5.0–7.0 months) than the other patients (*n* = 36/54; OS: 16.0 ± 2.4 months; 95% CI 11.2–20.8 months; *p* < 0.001; Fig. 2).

A significant difference in OS was also recorded between patients with (*n* = 23/54; 7.0 ± 2.7 months, 95% CI 1.6–12.4 months) and without visceral metastases (*n* = 31/54; 12.0 ± 2.1 months, 95% CI 7.9–16.1 months; *p* = 0.045) as well as between patients with liver metastases (*n* = 13/54; 3.0 ± 1.1 months, 95% CI 0.9–5.1 months) and without liver metastases (*n* = 41/54; 13.0 ± 2.2 months, 95% CI 8.7–17.3 months; *p* < 0.001). On univariate cox regression, the ECOG performance status (hazard ratio [HR]: 2.9 95% CI 1.7–5.0; *p* < 0.001) and the extent of metastases on PSMA ligand PET/CT (HR 2.3 95% CI 1.1.–4.8; *p* = 0.024) were also significant predictors of OS. Other factors (pretreatment with docetaxel or cabazitaxel, patient’s age, lines of pretreatment, time since initial diagnosis, and Gleason score) were not prognostic for OS (*p* > 0.1, respectively).

On multivariable analysis between FDG+/PSMA− lesions, visceral metastases, liver metastases, extent of metastases on PSMA ligand PET/CT, ECOG, pretreatment with docetaxel or cabazitaxel, patient’s age, lines of pretreatment, time since initial diagnosis, and Gleason score, FDG+/PSMA− lesions retained their prognostic capability regarding survival (HR 4.9 95% CI 1.7–14.3, *p* = 0.04) after stratification. Furthermore, ECOG performance status (HR 4.5 95% CI 1.8–11.6, *p* = 0.02) and the presence of liver metastases (HR 7.6 95% CI 1.2–49.3, *p* = 0.034) were also significant prognostic markers for OS. The other parameters were not prognostic for OS (*p* > 0.1).

## Discussion

Our analysis showed that FDG+/PSMA− lesions occurred in one third (33%) of patients with mCRPC prior to RLT, which is in line with a recent study by Wang et al. (33%) [19], and slightly higher compared to the results of Hofmann et al. (19%) [14]. FDG+/PSMA− lesions were found in skeleton or liver in 44% of cases, which was comparable to prior results in the study of Thang et al. (50% or 38%, respectively) [16]. Patients with FDG+/PSMA− lesions underwent significantly fewer cycles of RLT as these patients presented more often with a progression of disease under treatment. This could either be due to a missing response to PSMA RLT, or the consequence of a more aggressive disease. Presence of FDG+/PSMA− lesions could be confirmed as a negative prognostic factor: median OS for patients without discrepant FDG+/PSMA− lesions was significantly longer with 16 ± 2.4 months as compared to patients with FDG+/PSMA− lesions (6.0 ± 0.5 months). This finding is in line with the extended cohort of the LuPSMA Trial with an OS of 13.3 months [15]. Whereas patients with PSMA-negative viable diseases performed particularly poor, our results may indicate that RLT prolongs survival in these patients as compared to subjects being treated with best standard of care. In contrast to our approach in which patients with PSMA-negative metastases were offered PSMA-targeted RLT if this was their last therapeutic option and if the majority of metastases was PSMA-positive, the LuPSMA trial excluded subjects due to low-PSMA expression (8 of 16 patients) or FDG+/PSMA− findings (8 of 16 patients) [14, 15]. In this cohort, patients with FDG+/PSMA− lesions showed a poor median OS of only 3.9 months under standard of care, which is distinctly shorter than the comparable cohort in our study with 6.0 ± 0.5 months. Furthermore, the authors of the LuPSMA trial discuss that RLT might have improved survival also in this subcohort [16]. To our knowledge, this is the first analysis of patients with dual tracer imaging undergoing RLT without exclusion of patients with heterogeneous tumor lesions. Interestingly, the majority of patients with discordant sites of disease (15/18, 83%) had ≤ 3 FDG+/PSMA− lesions, though the impact of these single lesions was already distinct on OS as shown in a multivariable analysis also considering the extent of metastases on PSMA ligand PET/CT.

As further negative prognostic factors, poorer ECOG status and presence of liver metastases were confirmed on multivariable analysis. This is in line with previous findings demonstrating that the presence of visceral metastases, especially liver metastases, results in poorer survival [20] [21]. ECOG status has also been identified as a prognosticator of OS before [22]. The extent of metastases on PSMA ligand PET/CT was only a significant prognostic marker on univariate analysis.

### Limitations

This retrospective study suffers from some limitations, the first being the inclusion of a rather small, heterogeneous patient cohort. Second, PET/CT examinations were gained from both [^68^Ga] and [^18^F]-labeled PSMA ligands, as both hospitals adapted the radiolabeling procedure during the study period. In order to address potential differences of the PSMA-ligands, a robust visual categorization system with a substantial interrater agreement was developed. Similarly, the impact of different PET/CT scanners used at the two institutions seems negligible. Additionally, the influence of previous cytotoxic treatments can be considered rather small, as these treatments were stopped several weeks before PSMA ligand PET/CT and disease progression was documented.

Finally, the high physiological tracer uptake of the liver when using [^18^F]PSMA-1007 is a frequent and inherent limitation with possible false-negative hepatic lesions [23].

## Conclusion

This study demonstrates a significantly poorer response to PSMA-directed RLT in patients with mCRPC and FDG+/PSMA− lesions, which occurs in about one third of patients. These discordant findings probably indicate a more aggressive biology of disease, and it remains to be elucidated if this patient population still benefits from RLT. Therefore, prospective trials investigating optimal treatment protocols for patients with FDG+/PSMA− lesions, e.g. combination of PSMA-directed RLT and cytotoxic drugs or immunotherapy, are highly warranted.

## Supplementary Information

ESM 1 (DOCX 27 kb)

## Data Availability

Not applicable.
