# Supplementary material for: Prognostic implications of dual tracer PET/CT: PSMA ligand and [18F]FDG PET/CT in patients undergoing [177Lu]PSMA radioligand therapy
Source: Eur J Nucl Med Mol Imaging. 2020 Dec 18;48(6):2024–30. doi: 10.1007/s00259-020-05160-8 (PMC8113196; doi:10.1007/s00259-020-05160-8)
Supplement: Supplementary file 1 — (DOCX 27 kb) [file 259_2020_5160_MOESM1_ESM.docx]

**Tracer synthesis and PET/CT acquisition**

**Freiburg**:

Scans were either performed with a VEREOS Digital PET/CT or a GEMINI TF 16 Big-Bore PET/CT (both Philips Healthcare, USA). All patients were asked to void before PET. Images were reconstructed with a vendor-specific time-of-flight iterative reconstruction algorithm (BLOB-OS-TF) with 3 either iterations and 9 subsets (relaxation parameter 0.35) and a voxel size of 2 x 2 x 2mm³ (VEREOS Digital PET/CT) or with either 3 iterations and 33 subsets (relaxation parameter 0.35) and a voxel size of 2 x 2 x 2mm³ (GEMINI TF 16 BigBore PET/CT). The spatial resolution of the reconstructed PET image is about 5 mm (VEREOS) to 7 mm (GEMINI TF) full width half maximum (FWHM).

**Preparation of the radiopharmaceuticals:**

[^18^F]FDG, [^18^F]PSMA-1007 and [^68^Ga]Ga-PSMA-11 have been established for clinical routine applications in the department of nuclear medicine of the University hospital Freiburg.

[^68^Ga]Ga-PSMA-11:

PSMA-11 was labeled with [^68^Ga]Cl3 by using a fully-automated synthesis module according to Good Laboratory Practice in combination with sterile single-use cassettes (Eckert & Ziegler, Germany). The radiochemical purity of the final product was ≥97%. Injected activity of [^68^Ga]PSMA-11 was on average 206 MBq (range 204 – 211 MBq). At 1 hour after injection, patients underwent a whole-body PET (from skull to mid-thigh) with a scan duration of 2 minutes per bed position. A contrast-enhanced diagnostic CT (120 kVp, 100 - 400 mAs) with dose modulation was performed for anatomic correlation and attenuation correction.

[^18^F]PSMA-1007:

Radiosynthesis of [^18^F]PSMA-1007 was performed as described previously [1] on a GE TRACERlab MX (GE Healthcare, USA) or a NEPTIS Mosaic-RS (ORA, Belgium). The radiochemical purity of the final product was ≥91%. Injected activity of [^18^F]PSMA-1007 was on average 325 MBq (range 256– 454 MBq). At 2 hours after injection, patients underwent a whole-body PET (from skull to mid-thigh) with a scan duration of 2 minutes per bed position. A contrast-enhanced diagnostic CT (120 kVp, 100 - 400 mAs) with dose modulation was performed for anatomic correlation and attenuation correction.

[^18^F]FDG:

[^18^F]FDG was synthesized on a GE TRACERlab MX (GE Healthcare, USA). The radiochemical purity of the final product was ≥95%. Before [^18^F]FDG injection, patients had to fast for at least 6 hours and blood glucose level had to be lower than 150 mg/dL. Injected activity of [^18^F]FDG was on average 363 MBq (range 273 – 425 MBq). At 1 hour after injection, patients underwent a whole-body PET (from skull to mid-thigh) with a scan duration of 2 minutes per bed position. A low-dose CT (120 kVp, 25 mAs) was performed for anatomic correlation and attenuation correction.

**Würzburg:**

PET/CT Scans were either performed with a Siemens Biograph mCT 64 scanner or on a Siemens Biograph mCT 128 flow (Siemens, Knoxville, USA). All patients were asked to void before PET. All PET scans were acquired in three-dimensional mode. Images were reconstructed iteratively using an ordered subset expectation maximization algorithm with 3 iterations and 24 subsets followed by a post-reconstruction Gaussian filter smoothing (2mm fullwidth at half-maximum; Siemens TrueX) (Biograph mCT 64) or with 3 iterations and 21 subsets followed by a post-reconstruction Gaussian filter smoothing (2mm fullwidth at half-maximum; Siemens TrueX+TOF ultra HD)( Biograph mCT 128 flow). The spatial resolution of the reconstructed PET image is about 4.7 mm (Biograph mCT 128 flow) to 5.7 mm (Biograph mCT 64) full width half maximum (FWHM).

**Preparation of the radiopharmaceuticals:**

[^18^F]FDG, [^18^F]PSMA-1007 and [^68^Ga]Ga-PSMA I&T have been established for clinical routine applications in the department of nuclear medicine of the University hospital Wuerzburg.

[^68^Ga]Ga-PSMA I&T:

Gallium-68 **([**^68^Ga]) for radiolabeling was eluted with 0.1 M HCl in form of gallium-68 chloride (^68^GaCl_3_) from a [^68^Ge]/[^68^Ga]-generator (GalliaPharm, Eckart & Ziegler, Berlin, Germany). The synthesis of [^68^Ga]Ga-PSMA-I&T was carried out on a cassette-based synthesis module (Scintomics, Fürstenfeldbruck, Germany) as described previously [2]. The isolated product was formulated with an injectable phosphate buffer saline and passed through a 0.22 µm sterile filter into a sterile vial. The product was tested by TLC and HPLC for radiochemical purity as well as for sterility and endotoxins before human applications. Injected activity of [^68^Ga]Ga-PSMA I&T was on average 129 MBq (range 89 – 155 MBq). Variations in injected radiotracer activity were caused by the short half-life of ^68^Ga and variable elution efficiencies obtained during the lifetime of the [^68^Ge]/[^68^Ga] radionuclide generator. At 1 hour after injection, patients underwent a whole-body PET (from skull to mid-thigh) with a scan duration of 2 minutes per bed position. A contrast-enhanced diagnostic CT scan (100 - 120 kV, mA modulated) was performed for anatomic correlation and attenuation correction.

[^18^F]PSMA-1007:

Radiosynthesis of [^18^F]PSMA-1007 was performed as described previously [1] using a GE TRACERlab MX synthesis module (GE Medical Systems, Uppsala, Sweden). The radiochemical purity of the final product was ≥ 95 %. Injected activity of [^18^F]PSMA-1007 was on average 317 MBq (range 298 – 417 MBq). At 1.5 hours after injection, patients underwent a whole-body PET (from skull to mid-thigh) with a scan duration of 2 minutes per bed position. A contrast-enhanced diagnostic CT scan (100 - 120 kV, mA modulated) was performed for anatomic correlation and attenuation correction.

[^18^F]FDG:

[^18^F]FDG was produced using a GE Fastlab^®^ synthesis module (GE Medical Systems, Uppsala, Sweden). The radiochemical purity of [^18^F]FDG was ≥95%. Before [^18^F]FDG injection, patients had to fast for at least 6 hours and blood glucose level had to be lower than 180 mg/dL . Injected activity of [^18^F]FDG was on average 300 MBq (range 239 – 507 MBq). At 1 hour after injection, patients underwent a whole-body PET (from skull to mid-thigh) with a scan duration of 2 minutes per bed position. A low-dose CT (100 - 120 kV, 30 mAs) was performed for anatomic correlation and attenuation correction.

**References:**

1. Cardinale J, Martin R, Remde Y, Schäfer M, Hienzsch A, Hübner S, et al. Procedures for the GMP-Compliant Production and Quality Control of [(18)F]PSMA-1007: A Next Generation Radiofluorinated Tracer for the Detection of Prostate Cancer. Pharmaceuticals (Basel, Switzerland). 2017;10. doi:10.3390/ph10040077.

2. Hartrampf PE, Heinrich M, Seitz AK, Brumberg J, Sokolakis I, Kalogirou C, et al. Metabolic Tumour Volume from PSMA PET/CT Scans of Prostate Cancer Patients during Chemotherapy-Do Different Software Solutions Deliver Comparable Results? Journal of clinical medicine. 2020;9. doi:10.3390/jcm9051390.
